# A case of COVID‐19‐associated C‐ANCA vasculitis, was successfully treated with rituximab therapy

**DOI:** 10.1002/ccr3.9308

**Published:** 2024-08-10

**Authors:** Michael Filoramo, Renish Contractor, Deepak Chandramohan, Prabhat Singh, Nihar Jena, Prathap Kumar Simhadri

**Affiliations:** ^1^ FSU College of Medicine Daytona Beach Florida USA; ^2^ UAB School of Medicine Birmingham Alabama USA; ^3^ Christus Spohn Hospital Corpus Christi Texas USA; ^4^ Wayne State University Detroit Michigan USA

**Keywords:** ANCA‐vasculitis, ANCA vasculitis management, COVID‐19 induced ANCA vasculitis, COVID‐19 infection, infection associated vasculitis

## Abstract

Both C‐anti‐neutrophil cytoplasmic antibody (ANCA) and P‐ANCA vasculitis were reported to be associated with COVID‐19 infection. The ideal management of COVID‐19‐associated ANCA vasculitis is unclear, as the experiences were limited to case reports. We presented a case of COVID‐19‐associated C‐ANCA vasculitis, successfully treated with steroids and rituximab therapy without any significant adverse reactions.

## 1 INTRODUCTION

Anti‐neutrophil cytoplasmic antibody (ANCA) associated vasculitis is a group of systemic vasculitis that causes inflammation and destruction of the small and medium‐sized blood vessels. It is one of the major causes of rapidly progressive renal failure, and it carries the risk of significant morbidity and mortality. SARS‐CoV‐2 (COVID‐19) infection and COVID‐19 vaccination have been shown to increase the risk for autoimmune diseases. There are few case reports of ANCA vasculitis associated with COVID‐19 infection. Several types of systemic vasculitis, autoimmune arthritis, inflammatory myopathies, systemic lupus erythematosus, antiphospholipid antibody syndrome, and even sarcoidosis were reported in association with COVID‐19 infection.

Few cases of C‐ANCA and P‐ANCA vasculitis were reported in the literature. There is a lack of data regarding their ideal management. We present a case of an elderly female who developed C‐ANCA vasculitis a few weeks after her COVID‐19 infection, and she was successfully treated with steroids and rituximab.

## 2 CASE PRESENTATION

An 87‐year‐old white female with a history of hypertension and no other significant medical history presented to the emergency department complaining of worsening generalized weakness and tiredness for 2 weeks.

She tested positive for COVID‐19 infection after developing low‐grade fevers, generalized weakness, and dry cough symptoms about 3–4 weeks before the presentation. Those symptoms resolved spontaneously after a few days. She later started having worsening generalized weakness, tiredness, and diffuse muscle aches, which ultimately led to her current presentation. She received three doses of mRNA BNT162b2 vaccination; the last dose was given about 6 months before her presentation.

## 3 INVESTIGATIONS AND TREATMENT

She was noted to have abnormal renal parameters, with a creatinine (Cr) of 2.99 mg/dL, a BUN of 36 mg/dL, and an estimated GFR of 14 mL/min. The dipstick urine analysis showed 3+ proteinuria and 3+ hematuria. She had no history of kidney disease at baseline, and her baseline Cr was 0.90 mg/dL.

Serological workup showed negative ANA and no evidence of cryoglobulinemia, and urine and serum immunofixation showed no proof of paraproteinemia. She had normal complement levels with a C3 level of 120 and a C4 level of 29. She underwent 24‐hr urine collection, which was suggestive of 1.8 g of proteinuria. She had an abnormal elevation of serum Anti‐proteinase‐3 antibody titers of 1:946, elevated C‐ANCA titers with 1:1280, and tested negative for anti‐myeloperoxidase (MPO) and P‐ANCA antibodies, as shown in Table 1. Before this presentation, she had never had any autoimmune titers done that could be used for comparison, as she had no symptoms suggestive of an autoimmune process. She underwent a CT of the chest, which was suggestive of a 3 cm pulmonary mass in the right upper lobe.

**TABLE 1:** Metabolic profile, urinalysis, and autoimmune titers.

Lab	On admission November/2022	On follow‐up April/2023	On follow‐up Sep/2023	Normal values
Na	137 mmol/L (137 mmol/L)	141 mmol/L (141 mmol/L)	137 mmol/L (137 mmol/L)	135–145 mmol/L (135–145 mmol/L)
K	4.1 mmol/L (4.1 mmol/L)	4.6 mmol/L (4.6 mmol/L)	3.7 mmol/L (3.7 mmol/L)	3.4–5.0 mmol/L (3.4–5.0 mmol/L)
Cl	102 mmol/L (102 mmol/L)	104 mmol/L (104 mmol/L)	101 mmol/L (101 mmol/L)	98–107 mmol/L (98–107 mmol/L)
C02	24.0 mmol/L (24.0 mmol/L)	23 mmol/L (23 mmol/L)	25 mmol/L (25 mmol/L)	22.0–29.0 mmol/L (22.0–29.0 mmol/L)
BUN	**36.0 mg/dL** **(12.85 mmol/L)**	**43.0 mg/dL** **(15.56 mmol/L)**	**26.8 mg/dL** **(9.75 mmol/L)**	8–23 mg/dL. (2.9–8.9 mmol/L)
Cr	**2.99 mg/dL** **(264.4 umol/L)**	**3.00 mg/dL** **(251.11 umol/L)**	**2.12 mg/dL** **(187.45 umol/L)**	0.51–0.95 mg/dL (44‐97umol/L)
Glucose	89 mg/dL (4.94 mmol/L)	81 mg/dL (4.49 mmol/L)	84 mg/dL (4.66 mmol/L)	70–99 mg/dL (3.89–5.5 mmol/L)
Ca	9.4 mg/dL (2.35 mmol/L)	9.1 mg/dL (2.27 mmol/L)	**8.4 mg/dL** **(2.1 mmol/L)**	8.8–10.2 mg/dL (2.2–2.54 mmol/L)
MPO	0 AU/mL	0 AU/mL	0 AU/mL	0–19 AU/mL
Serine Protease 3 Ab	**946 AU/mL**	**252 AU/mL**	**112 AU/mL**	0–19 AU/mL
C‐ANCA	**>1:1280**	**1:80**	<1:20	<1:20
Glucose Qual, Urine	Negative	Negative	Negative	Negative
Protein Qual, Urine	**3+**	**1+**	**1+**	Negative
Bilirubin, Urine	Negative	Negative	Negative	Negative
pH, Urine	5.5	5.0	5.5	5.0–8.0
Blood, Urine	**3+**	**1+**	**1+**	Negative
RBC	> 30/HPF	3/HPF	8/HPF	Negative
Nitrite, Urine	Negative	Negative	Negative	Negative
Leukocyte Esterase, Urine	**Trace**	**Negative**	Negative	Negative
Specific Gravity, Urine	1.018	1.014	1.014	1.005–1.030
C3 Complement	120 mg/dL	106 mg/dL	106 mg/dL	90–180 mg/dL
C4 Complement	29.0 mg/dL	29.3 mg/dL	29.3 mg/dL	10.0–40.0 mg/dL

*Note*: Abnormal results are highlighted in bold.

Abbreviations: ANCA, anti‐neutrophil cytoplasmic antibody; BUN, blood urea nitrogen; Ca, calcium; Cl, chloride; CO_2_, bicarbonate; Cr, creatinine; HPF, high power field; K, potassium; MPO, anti myeloperoxidase antibody; Na, sodium; RBC, red blood cells.

**FIGURE 1:**
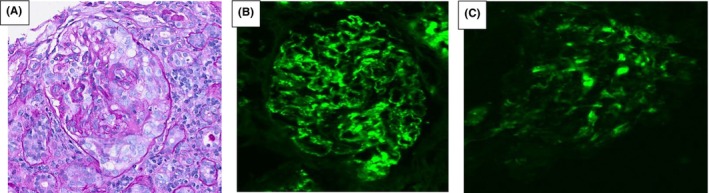
(A) The H&E stain showing segmental fibrocellular crescent with moderate mixed interstitial inflammatory infiltrate. (B) The 3+ Ig G staining on immunofluorescence. (C) The 2+ C3 staining on immunofluorescence.

Renal biopsy was suggestive of necrotizing and crescentic glomerulonephritis. Three of the 11 non‐globally sclerotic glomeruli showed necrosis with crescent formation. Immunofluorescence showed granular mesangial and capillary loop staining with positive staining for IgG, IgM, C3, kappa, and lambda, as shown in Figure 1. Electron microscopy revealed numerous subepithelial, intramembranous, and segmental subendothelial electron‐dense deposits and basement membranes.

Negative blood and urine cultures ruled out infection‐related crescentic vasculitis. She did not have any vegetation on the transthoracic echocardiogram. She was given pulse dose steroids, started on Rituxan 375 milligrams/meters squared dosing, sulfamethoxazole/trimethoprim for PCP prophylaxis, and continued on losartan. She received four weekly doses of Rituxan, her steroids were tapered to 10 mg daily over 8 weeks, and she was maintained on prednisone 5 mg daily for a total of 52 weeks.

## 4 OUTCOME AND FOLLOW‐UP

**GRAPH 1:**
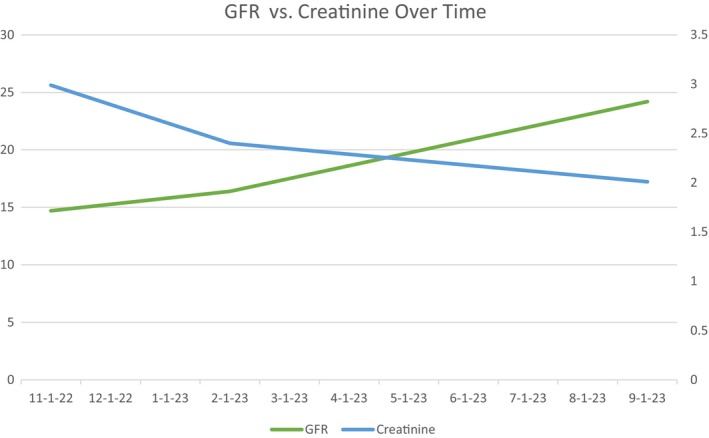
GFR values are mentioned on the left side and the creatinine (Cr) values are on the right side of the graph. All Cr values reported in mg/dL. All GFR values reported in mL/min/1.73m^2^. Graph shows the trends of serum Cr and eGFR over time. eGFR values are mentioned on the left side of the *y*‐axis, and Cr values are on the right side. The time frame of those labs is mentioned on the *x*‐axis.".

Her symptoms improved, including generalized weakness, anemia, and diffuse muscle aches. A follow‐up imaging study of the right lung showed the resolution of the right lung mass. Her Cr has improved to 2.4 with an eGFR of 17, as shown in Graph 1. Her proteinuria, hematuria, and C‐ANCA titers have also improved. She is being continued on 500 mg of IV Rituxan every 6 months for maintenance.

## 5 DISCUSSION

ANCA‐associated vasculitides are relatively rare autoimmune conditions that cause inflammation of blood vessels, which can manifest in various forms. The three main types of ANCA‐vasculitides are granulomatosis with polyangiitis (GPA), eosinophilic granulomatosis with polyangiitis, and microscopic polyangiitis. The incidence of these vasculitides is approximately 10–20 cases per million, with GPA being the most common type of small vessel vasculitis.^1^ Typical presentations of ANCA‐associated vasculitides include fatigue, body aches, muscle pain, cough, fever, abdominal pain, and hemoptysis. Renal manifestations can present with hypertension, new onset proteinuria, hematuria, and active urinary sediments and can progress rapidly to renal failure. It has been shown that approximately 90% of patients with GPA have ANCA positivity, as it was positive in this patient's presentation.^1^

As we continue to learn more about COVID‐19, there has been an increasing awareness amongst the medical community that this virus influences a wide array of body systems, manifesting as a constellation of symptoms. Multiple studies conducted across different patient populations have highlighted that there is a significant elevated risk of autoimmune and autoinflammatory disorders after COVID‐19 infection.^2^, ^3^ Autoimmune and autoinflammatory conditions such as immune thrombocytopenia, psoriasis, IgA nephropathy, systemic lupus erythematosus, myocarditis, and others have been reported in the literature shortly after COVID‐19 infection.^4^ A few cases of ANCA vasculitis were reported after COVID‐19 infection.^5^, ^6^

There were cases of autoimmune phenomenon documented with the COVID‐19 vaccination as well.^7^ The COVID‐19 vaccination in our patient could have also triggered the onset of C‐ANCA vasculitis. Still, the stronger association is with the infection as the timeline between the onset of renal failure and the infection was about 6 weeks. In contrast, the timeline between the last dose of vaccination and the development of renal failure was more than 6 months.

The mainstay of treatment of ANCA‐associated vasculitides focuses on slowing further organ involvement, typically with a combination of corticosteroids with cyclophosphamide or rituximab. Maintenance of remission is generally achieved with an 18–24‐month course of methotrexate, mycophenolate mofetil, or azathioprine.^1^ The treatment of ANCA vasculitis has evolved from cyclophosphamide to rituximab over recent years, as multiple randomized trials showed rituximab therapy as non‐inferior to cyclophosphamide‐based therapy.^8^, ^9^ Recent literature also suggested that maintenance therapy with rituximab is superior to oral azathioprine therapy.^10^

There is insufficient literature to direct the appropriate treatment of ANCA vasculitis associated with COVID‐19 infection. A few case reports used a combination of cyclophosphamide and steroids, and a few others used a combination of rituximab and steroids with variable success.^5^, ^6^ We were able to successfully achieve remission in our case by using a combination of rituximab and steroids. She was maintained on low‐dose prednisone therapy for 52 weeks based on evidence that it decreases the risk of disease flare‐ups.^11^ She was kept on sulfamethoxazole/trimethoprim therapy for Pneumocystis prophylaxis for 6 months. She also had significant improvement in her constitutional symptoms, anemia, proteinuria, and hematuria, and she never required renal replacement therapy. We plan to continue administration of Rituximab 500 mg dose every 6 months to prevent future recurrences.

## 6 CONCLUSION

This case of ANCA‐associated vasculitis shortly after COVID‐19 infection demonstrates that healthcare professionals must recognize that COVID‐19 can be associated with the onset of ANCA‐associated vasculitis and other autoimmune conditions. These patients can present with non‐specific symptoms, and we should have a high index of suspicion. This case also highlights that a combination of rituximab and steroids is an effective and safe therapy for COVID‐19‐associated ANCA vasculitis.

## AUTHOR CONTRIBUTIONS

**Michael Filoramo:** Writing – original draft. **Renish Contractor:** Writing – original draft. **Deepak Chandramohan:** Supervision; writing – review and editing. **Prabhat Singh:** Supervision; writing – review and editing. **Nihar Jena:** Writing – original draft; writing – review and editing. **Prathap Kumar Simhadri:** Conceptualization; supervision; writing – review and editing.

## FUNDING INFORMATION

No funding was needed to prepare this manuscript.

## CONFLICT OF INTEREST STATEMENT

All authors do not have any financial or non‐financial conflicts of interest concerning this submission.

## CONSENT

Written informed consent was obtained from the patient to publish this report in accordance with the journal's patient consent policy.

## DISCLOSURE

This case was presented as an abstract at the American Society of Nephrology 2023 meeting.

## Data Availability

Data sharing does not apply to this article as no new data were created or analyzed in this study.
